# The effect of combining primers and cements from different cement systems on the bond strength between zirconia and dentin

**DOI:** 10.1038/s41405-024-00230-7

**Published:** 2024-06-05

**Authors:** Minh Le, Evaggelia Papia, Christel Larsson

**Affiliations:** 1 Department of Prosthodontics, Faculty of Odontology, Malmö University, Malmö, Sweden; 2 Department of Materials Science and Technology, Faculty of Odontology, Malmö University, Malmö, Sweden; 3 Department of Prosthodontics, Riga Stradins University, Riga, Latvia

**Keywords:** Composite resin, Dentistry

## Abstract

**Objective:**

The aim of this study was to evaluate the influence of combining primers and cements from two different resin cement systems on the microtensile bond strength (μTBS) between zirconia and human dentin.

**Materials and methods:**

A total of 120 specimens of zirconia cemented to dentin were allocated into eight groups based on cement type (RelyX Ultimate or Panavia V5) and primers (Tooth Primer, Clearfil Ceramic Primer and Scotchbond Universal Adhesive) combinations, applied to dentin or ceramic surfaces. Following artificial aging with 5000 thermocycles, μTBS tests were conducted. Statistical analysis was performed using One-way ANOVA and Tukey’s post hoc tests (*p* ≤ 0.05), and failure modes were assessed.

**Results:**

The Panavia V5 cement system demonstrated the highest bond strength (19.4 ± 4.4 MPa), significantly higher than the other groups except when RelyX cement was used with Panavia primers (16.9 ± 3.7 MPa). Cohesive fractures within the cement layer were the predominant failure mode.

**Conclusions:**

The combination of primers from different adhesive cement system brands may significantly affect the bonding effectiveness. Therefore, using products from a single product line of the same adhesive cement system, and following the manufacturer’s recommendations for indications and use, is crucial for a more predictable clinical outcome.

## Introduction

All-ceramic restorations are appreciated for their biocompatibility and esthetics and stand out as one of the most popular restorative materials [1, 2]. While restorations made of zirconia have demonstrated high survival rates, failures such as loss of retention still occur [3, 4]. Due to their lack of a glass phase, these materials are not etchable like glass ceramics, which limits their ability to achieve a strong bond [5, 6]. However, in vitro studies have provided evidence of the potential to establish a durable bond to zirconia [7, 8]. These studies highlight the importance of restoration pretreatment, particularly airborne abrasion. Furthermore, adhesive cement systems incorporating a specific phosphate monomer component, 10-methacryloyloxydecyl dihydrogen phosphate (10-MDP), either in the primer or in the cement, have been shown to enhance adhesion to zirconia [5, 9, 10]. Alternative treatments such as acid etchants or plasma coating of zirconia surfaces have been suggested [6, 8]. However, the feasibility of these treatments in daily clinical practice is limited because of the higher complexity and costs of these techniques [7]. The tooth–cement interface is also of particular importance for zirconia-based restorations, especially when retention primarily relies on adhesive bonding [7]. Enamel adhesion, characterized by strong bonding between the enamel and adhesive resin, is regarded as reliable [11], whereas establishing a durable bond with dentin continues to be a challenge [12, 13].

Advancements in adhesive dentistry provide the general practitioner with a broad selection of adhesive cement systems [14]. To simplify daily practice, clinicians often favor “universal” adhesives, valuing their relative simplicity and compatibility with direct composite materials as well as indirect ceramic restorations [11, 15]. Nevertheless, some clinicians may prefer different cements for different types of restorations. As a result, the practice of combining products from different brands may have become more common [16]. However, manufacturers recommend only using components within the same cement systems for restorations due to tailormade combinations of chemical composition and compatibility. For instance, certain combinations of primer and resin cement may not be compatible and could potentially compromise bonding effectiveness [17, 18]. Strictly following the manufacturers’ recommended indications and instructions may have a critical importance on the clinical outcome of a bonded restoration; however, this issue has not been fully investigated [6, 14, 15, 18].

The aim of the present study was therefore to assess the effect of combinations of primers and cements from two different commercial resin cement systems on the microtensile bond strength (μTBS) between zirconia ceramic and human tooth tissue (dentin). The research hypothesis tested was that, compared to using the manufacturer-recommended products all together (controls), combinations of bonding products between the two commercial systems would result in significantly affect bond strength values.

## Materials and methods

Extracted human premolars and molars were collected after obtaining informed consent of the patients. Ethical vetting was obtained from Swedish Ethical Review Authority (DNR 2021/03119).

**Fig. 1:**
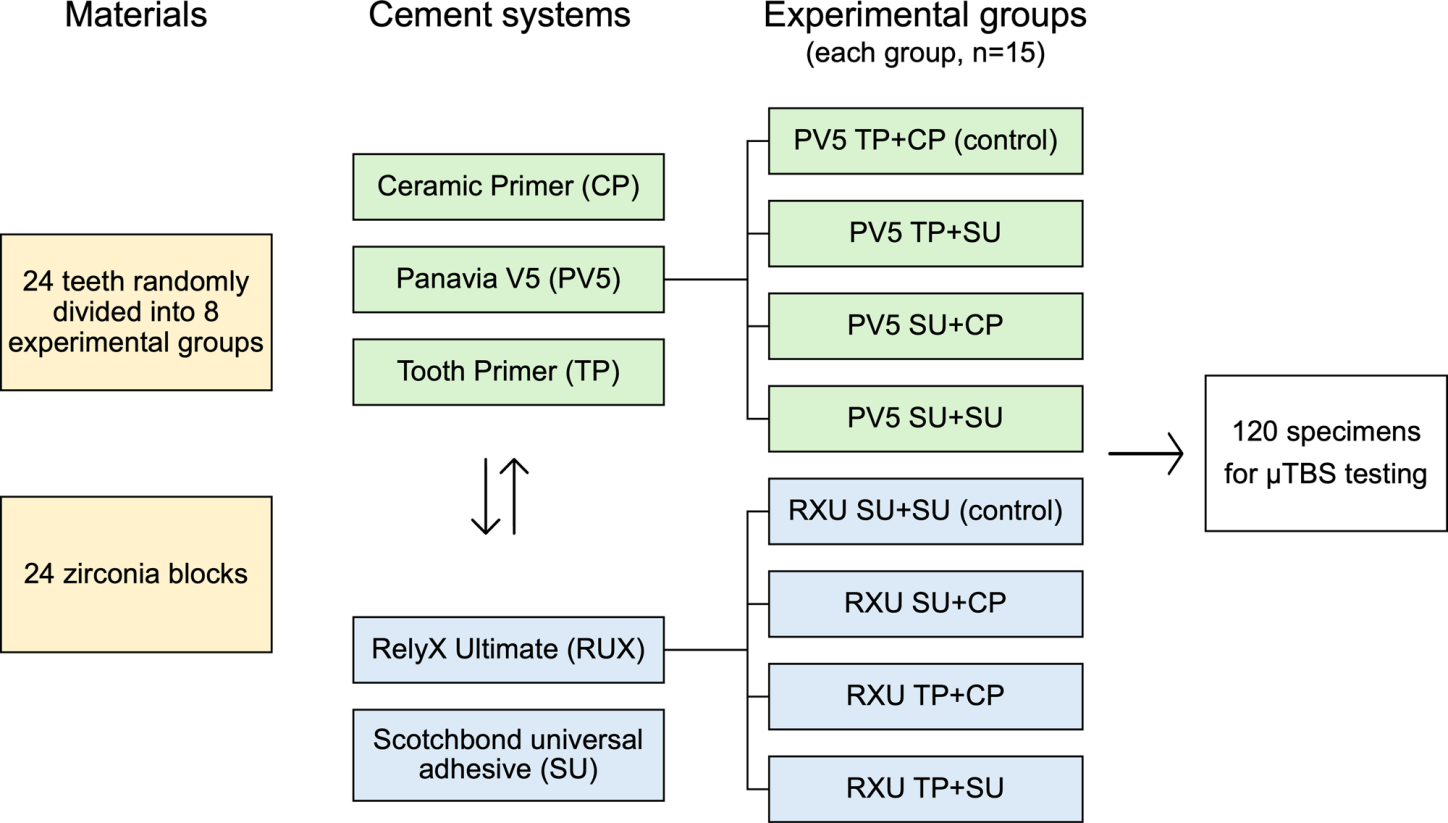
Study design. PV5 Panavia V5, TP Tooth Primer, CP Clearfil Ceramic Primer Plus, RXU RelyX Ultimate, SU Scotchbond Universal Adhesive, Control manufacturer’s components.

Twenty-four teeth were randomly divided into eight groups, with three teeth in each, based on the experimental conditions, Fig. 1. After removal of debris and any remaining soft tissue, the teeth were stored in a 70% ethanol solution at a temperature of 5 °C prior specimens’ preparation. A total of 120 specimens underwent a microtensile bond strength (μTBS) test within one month following tooth extraction. Two types of adhesive cement systems (RXU, RelyX Ultimate, 3M Deutschland, Neuss, Germany, and PV5, Panavia V5, Kuraray Noritake Dental, Tokyo, Japan) were used. Table 1 provides details about the two adhesive cement systems.

**Table 1:** Material compositions of the adhesive cement systems (obtained from manufacturer’s information and safety data sheets 2024).

Materials (Batch #)	Abbreviation	Manufacturer	Composition
Panavia V5 paste (9H0173)	PV5	Kuraray Noritake Dental, Japan	Bis-GMA (5–15%), TEGDMA (<5%), titanium oxide (<5%), CQ, hydrophobic aromatic dimethacrylate hydrophilic aliphatic dimethacrylate, silanated barium glass filler, silanated fluoroalminosilicate glass filler, silanated alumina filler, amorphous silica, accelerators, initiators, pigments
Tooth Primer (230087)	TP	Kuraray Noritake Dental, Japan	10-MDP, HEMA (25–50%), hydrophilic aliphatic dimethacrylate, accelerators, water pH value: <2.3
Clearfil Ceramic Primer Plus (2A0061)	CP	Kuraray Noritake Dental, Japan	10-MDP, ethanol (>80%), 3-trimethoxysilylpropyl methacrylate (<5%),
RelyX Ultimate (9636076)	RXU	3M Deutschland, Germany	TEGDMA, silane treated glass powder (50-60%), phosphorylated methacrylate (20-30%), phosphorylated methacrylate (20-30%), sodium persulfate, silane treated silica, perester, glass powder, acetic acid, copper salt, monohydrate
Scotchbond universal adhesive (30125B)	SU	3M Deutschland, Germany	MDP, HEMA, dimethacrylate resins, vitrebond copolymer, filler, ethanol, water (10-15%), silane (<5%), initiators pH value: 2.7

*Bis-GMA* bisphenol A diglycidylmethacrylate, *TEGDMA* triethyleneglycol dimethacrylate, *CQ* dl-camphorquinone or camphorquinone, *HEMA* 2-hydroxyethylmethacrylate, *10-MDP* 10-methacryloyloxydecyl dihydrogen phosphate.

### Specimen preparation

Following collection, specimen preparation involved several steps and followed a standardized protocol [19]. Prior to preparation, the extracted teeth were stored in distilled water at (23 ± 2) °C for a minimum of 12 h to remove residual ethanol. The teeth were then rinsed under running water for 30 min. Each tooth was mounted in a mold and secured above the level of the root using a cold-curing resin (VariDur 200, Beuhler, Lake Bluff, IL, USA). Occlusal enamel was removed perpendicular to the long axis of the tooth to expose a flat dentin surface using a diamond disc under water lubrication (IsoMet® 5000 Linear precision saw, Beuhler, Lake Bluff, IL, USA). Exposed dentin was dried with oil-free air and checked for enamel remnants using a light microscope. Following this treatment, a standardized smear layer was created by wet grinding the dentin surface using 600-grit size silicon carbide paper for 60 s at 250 rpm in a circular motion (Carbimet SiC Abrasive Paper, Beuhler, Lake Bluff, IL, USA). Zirconia ceramic blocks (KATANA Zirconia STML, A2, Kuraray Noritake Dental, Tokyo, Japan), with dimensions of 8 × 8 mm and 6 mm in height, were fabricated. These were milled and sintered by an authorized commercial dental laboratory following the manufacturer’s recommendations. The pretreatment of the zirconia surface also adhered to manufacturer’s recommendations. This treatment involved air abrasion using 50 µm aluminum oxide particles (Cobra, Renfert, Hilzingen, Germany) for 10 s, at a pressure of 2 bars at a distance of 10 mm. During this process, the nozzle was gently moved perpendicular to the surface. All zirconia surfaces were thoroughly washed using water and air-dried. The dentin was gently dried with oil-free air spray, leaving the surface visibly moist prior to cementation.

The specific primers were applied to the flat dentin or to the zirconia surfaces according to the manufacturer’s instructions. Regarding PV5 cement system, the ceramic primer (CP) was applied on the zirconia surface and dried. For the dentin, the tooth primer (TP) was applied, left for 20 s, and then thoroughly dried with oil-free air spray. The RelyX cement system has the same procedure for both dentin and zirconia surfaces: the primer (SU) was applied for 20 s and then thoroughly dried with oil-free air spray.

After applying the cement to the cementation surfaces of dentin and zirconia, a seating load of 15N was applied and maintained during the cementation procedure. Excess resin was removed from the margin using disposable brushes prior to polymerization and light-curing (Bluephase PowerCure, Ivoclar Vivadent, Schaan, Liechtenstein). The radiant exitance of this unit was 1200 mW/cm. The cement interface of the specimens was photopolymerized uniformly for 20 s on each side. Cementation was carried out using a randomized, group-by-group basis. All steps were performed by the same operator.

Bonded specimens were subsequently stored in water for at least 2 h before they were further sectioned. Subsequent sectioning was performed using a low-speed diamond disc under water irrigation along two axes (x and y) vertically into serial slabs resulting in a stick shape (IsoMet 5000 Linear precision saw, Series 15 LC Diamond, Beuhler, Lake Bluff, IL, USA). The initial section was discarded due to the probability of the presence of enamel and cement discrepancies, and only the inner sticks were included in the study. The cross-sectional area of the stick specimen was examined using a light microscope (Wild M3, Wild Heerbrugg, Heerbrugg, Switzerland), and only those with intact cementation joint and that were free of visible defects were included. Non-trimmed stick specimens with a bonded area of 1.0 ± 0.1 mm^2^, as measured with a digital caliper, were produced. Approximately 5–10 sticks were fabricated from each tooth, and the specimens were kept wet throughout the preparation procedures.

All specimens underwent artificial aging, consisting of 5000 thermocycles (Thermocycler 1100/1200, SD Mechatronik, Germany), between two temperature-controlled water baths: one at 5 °C and one at 55 °C. Each cycle lasted 60 s, with 20 seconds dwell time in each bath and 10 s for transfer between the baths. Following thermocycling, the specimens were stored in water at room temperature (22 °C) for up to 24 h prior to the μTBS test.

### Micro-tensile bond strength (μTBS) testing

A total of 120 specimens, were allocated into eight groups, for μTBS testing. Any specimens that failed prior testing were discarded, and other specimens were replaced until 15 successful tests were attained in each group. Each specimen was carefully positioned in the tensile tester (Type 4465, Instron, Canton, MA, USA), with the ends of each stick fixed to the device using a flowable composite resin (Tetric EvoFlow, Ivoclar Vivadent, Schaan, Liechtenstein). The crosshead speed was 0.75 mm/min, and the load at fracture was recorded (in N). Bond strength values were presented in MPa, calculated by dividing the tensile force at the time of fracture by the bonded surface area (in mm²). Mean and standard deviations for each group were calculated from the data collected.

### Failure mode

Following μTBS tests, all specimens were visually examined under a light microscope (Wild M3, Wild Heerbrugg, Switzerland) at ×30 magnification to classify failure modes as follows: cohesive fracture in dentin, cohesive fracture in zirconia, or cohesive fracture within cement, or adhesive failure to dentin, zirconia, or to both surfaces (mixed).

### Statistical analysis

The determined number of specimens in each group was in accordance with a standardized protocol [19]. Statistical analysis was conducted using statistical software (IBM SPSS Statistics, version 29, IBM, Chicago, IL, USA). The significance level was set α of 0.05. Normal distribution of the data was assessed using the Kolmogorov–Smirnov test. One-way analysis of variance (ANOVA) using Tukey’s HSD post hoc test to identify differences in bond strength between the groups, while Pearson’s Chi-square test was applied to evaluate differences among failure modes.

## Results

The mean μTBS values for each group are presented in Table 2. The Panavia V5 cement system (manufacturer’s components, control) demonstrated significantly higher bond strength (PV5 TP + CP: 19.4 ± 4.4 MPa) compared to other groups (*p* < 0.001), except for RelyX cement when combined with Panavia’s primers (RXU TP + CP: 16.9 ± 3.7 MPa), where the difference was not statistically (*p* = 0.378).

**Table 2:** Mean μTBS values (±standard deviations) in MPa.

#	Groups	Mean ± SD	Specimens, *n* (PTF)	Teeth
1	PV5 TP + CP control	19.4 ± 4.5 (a)	15 (2)	3
2	PV5 SU + CP	13.9 ± 3.3 (b, c)	15 (0)	3
3	PV5 TP + SU	11.8 ± 3.0 (c, d)	15 (1)	3
4	PV5 SU + SU	9.9 ± 3.3 (d, e)	15 (1)	3
5	RXU SU + SU control	9.6 ± 2.7 (d, e)	15 (1)	3
6	RXU SU + CP	8.5 ± 2.1 (d, e)	15 (2)	3
7	RXU TP + SU	8.2 ± 2.3 (e)	15 (0)	3
8	RXU TP + CP	16.9 ± 3.7 (a, b)	15 (2)	3

*PV5* Panavia V5, *TP* Tooth Primer, *CP* Clearfil Ceramic Primer Plus, *RXU* RelyX Ultimate, *SU* Scotchbond Universal Adhesive, *Control* manufacturer’s components, *PTF* Number of pretest failures.

Values represented by the same letter are not significantly different at *p* > 0.05.

In contrast, combining Scotchbond Universal Adhesive with the primers from the Panavia cement system did not significantly affect the bond strength of RelyX cement system. However, an exception was observed: the combination of RelyX cement with Panavia V5 primers significantly improved bond strength (*p* < 0.001).

### Failure mode

**Fig. 2:**
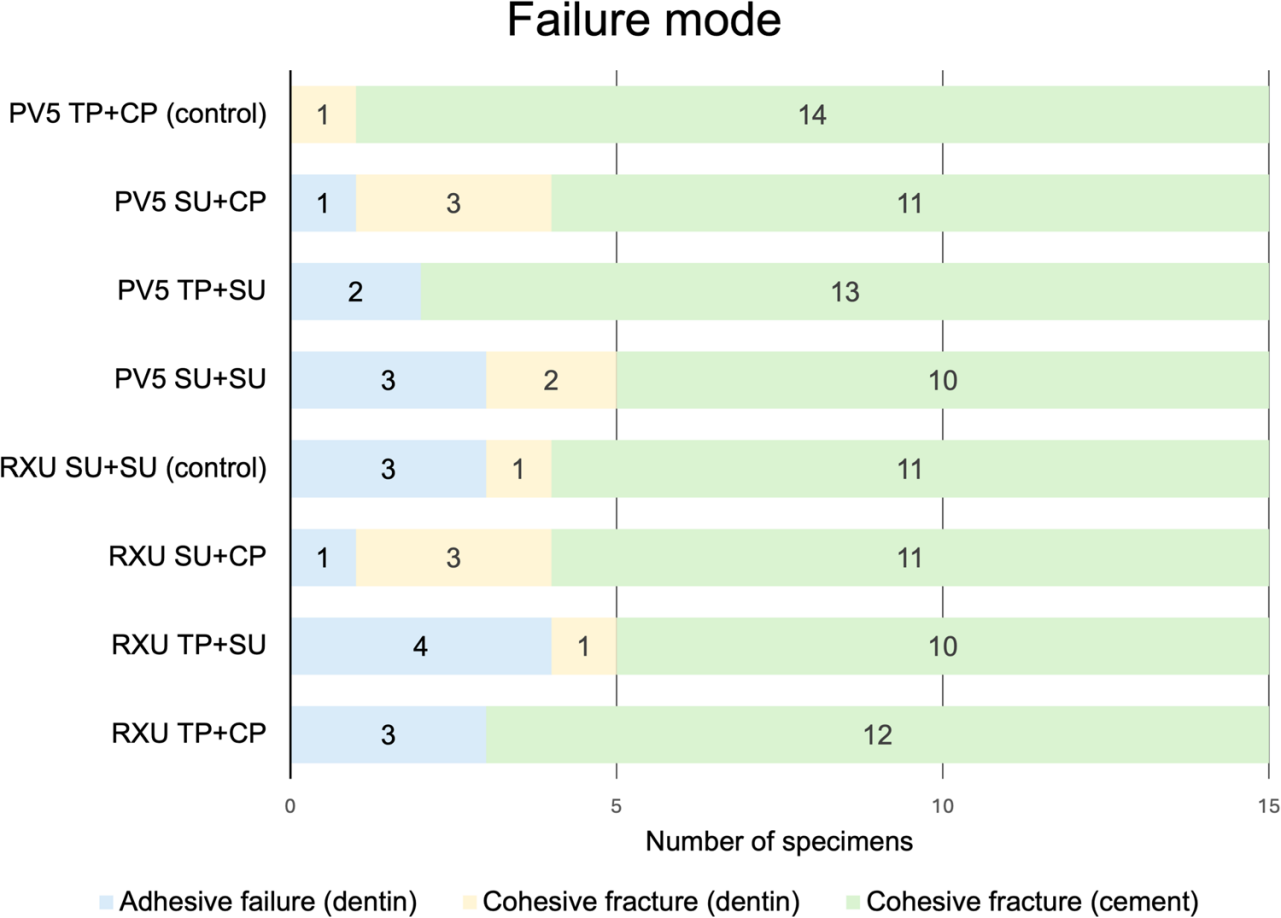
Failure mode distribution of specimens. PV5 Panavia V5, TP Tooth Primer, CP Clearfil Ceramic Primer Plus, RXU RelyX Ultimate, SU Scotchbond Universal Adhesive, Control manufacturer’s components.

**Fig. 3:**
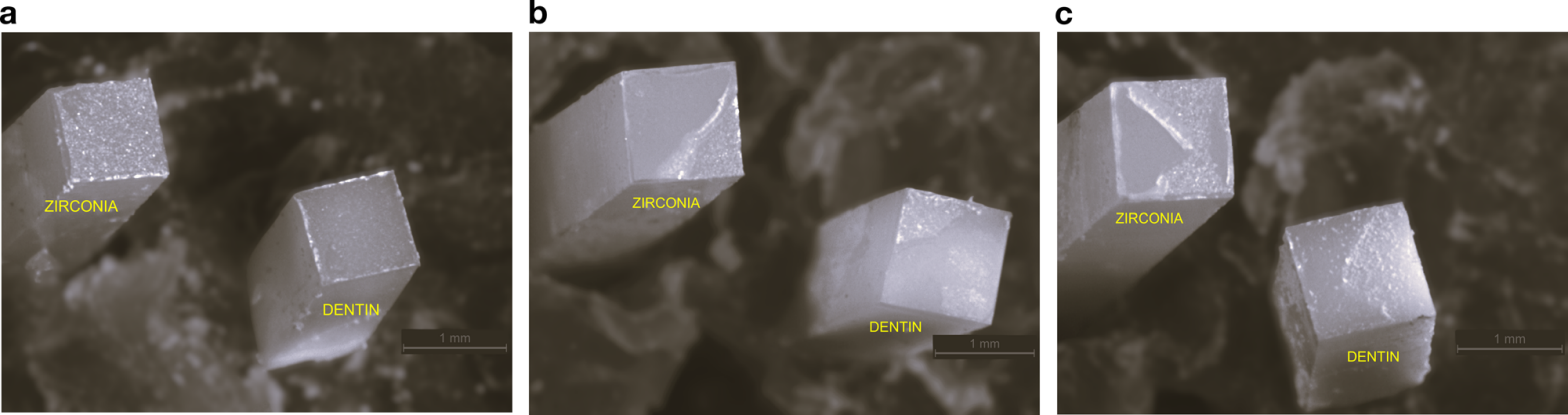
Representative microscopical images of fractured surfaces. **a** Adhesive failure, **b** cohesive fracture (dentin), **c** cohesive fracture (cement).

The failure mode distribution of specimens is reported in Fig. 2. All fractures occurred either within the cement or dentin (cohesive fracture) (Fig. 3a, b). No adhesive failures or cohesive fractures were detected on the zirconia surfaces. However, adhesive failures were found on dentin in all groups (Fig. 3c), except for group PV5 TP + CP, which showed none.

## Discussion

The research hypothesis, which suggested that combining different primers and cements from various systems would affect the bond strength between zirconia and a dentin surface, was accepted. The results show that not only do these combinations not necessarily decrease bond strength but also enhance it in some cases. Primers from the PV5 cement system demonstrated the highest bond strength to zirconia compared to other primer combinations, regardless of the type of resin cement used. This superior performance may be attributed to the fact that these primers are specifically developed for zirconia, while SU is marketed as a multipurpose primer with a formulation and composition that might differ from the specialized primers for zirconia [11, 14]. One significant difference between these cement systems lies in the pH value of the primers for the tooth surface: Scotchbond universal primer is considered an ultra-mild etchant (pH > 2.5), whereas Panavia tooth primer is a mild etchant (pH ≈ 2). Mild primers are preferable to ultra-mild primers, as they have shown better efficacy in removing smear layers and demineralizing the superficial layer of the dentin surface. This increased etching potential, in turn, can enhance bonding effectiveness, as surface smear may interfere with the bonding process [11].

All primers used in the present study contained the key component 10-MDP, which has been recommended to achieve a durable bond to zirconia [2, 7, 8]. However, the bonding effectiveness is not only dependent on the presence of these monomers but also on parameters such as the concentration and quality (purity) of the 10-MDP in the adhesive material [11]. In vitro studies show that impurities of this monomer can inhibit the chemical interaction of 10-MDP with hydroxyapatite of tooth tissue, which may negatively influence the bond strength [9]. The Panavia cement system containing the functional monomer 10-MDP has been shown to have a superior purity grade compared to other commercial primers [9]. Furthermore, the concentration of 10-MDP has been confirmed to significantly enhance the bond strength to zirconia. However, detailed information on the exact concentration and quality of the chemicals used in the present study are not disclosed, as manufacturers commonly consider this information to be a trade secret [6, 11].

Thermocycling was used to stress the bonded interface of the specimens, simulating an intra-oral aging scenario. In the absence of consensus on aging procedures, particularly in the context of evaluating adhesion to oxide ceramics with resin cement in laboratory settings, a minimum of 5000 thermocycles is considered sufficient [6, 10]. Given that the bonded area of the specimens was ~1 mm², a more pronounced aging effect, such as hydrolysis of the cement interface, could be expected [6]. The hydrolysis effects of thermocycling significantly affect bond strength of resin cement [8, 20]. Interestingly, bond strength values obtained using the Panavia adhesive cement systems have been shown to be stable and modestly affected by thermocycling procedures [21–23]. One possible explanation for this resilience could be that the monomer in these cement systems is less sensitive to hydrolysis due to the specific structures of their 10-MDP formulation [9]. In summary, these factors could explain the superior performance of Panavia cement system as observed in the present study.

The μTBS test was used as it is considered a versatile and standard method for testing bond strength [5, 6, 13, 24]. An advantage of this test method is that it focuses on clinically relevant substrates, and it requires less material to produce specimens compared to shear bond strength tests. Furthermore, the test promotes a more homogeneous stress distribution at the bonded interface [25]. Unlike the shear bond strength test, failures in this method often originate in the adhesive zone rather than within the bonded substrates [25]. In the present study, specimens were prepared using the non-trimmed technique in a rectangular cross-sectional shape, which is considered easier and less technique-sensitive compared to the preparation of cylindrical cross-sectional specimens. The importance of the geometry of these specimens has been emphasized, where cylindrical cross-sectional specimens are favored because they theoretically provide a more uniform stress distribution along the resin–dentin interface, in contrast to the rectangular cross-sectional specimens [13]. However, when comparing the performance of these cross-sectional shapes, similar bond strengths have been observed [26, 27]. This finding suggests that the non-trimming technique could be a viable, simpler alternative for specimen preparation in an already complex procedure.

Cohesive fractures within the cement were predominant in all groups, regardless of the cement system or cementation procedure. Fracture occurrence within the interface is preferable, as it is considered to accurately represent the actual bond strength of the cement. Reports indicate that in bond strength tests, the modes of failure primarily show cohesive fractures occurring more frequently in resin composite or ceramic materials than at the cement interface [24]. In μTBS tests, cohesive failures occur predominantly in the cement layer, contrary to shear bond tests. Therefore, μTBS tests have been considered more appropriate for evaluating the bond strength of resin composite to ceramic [25]. The findings might thus more accurately reflect the bond strength in the bonding area rather than the strength of the test materials themselves. No cohesive fractures or adhesive failure in zirconia were observed. This finding is in line with previous studies where pretreatment with airborne abrasion, in combination with a 10-MDP based adhesive cement, leads to durable bonding to zirconia [7, 8, 10]. However, several cohesive fractures and adhesive failures were noted on the dentin surface. Establishing a durable bond to enamel has been proven to be reliable, in contrast to dentin, which is acknowledged to be more challenging [11, 13]. The variability in dentin structure, such as differences in hydroxyapatite content and humidity, proximity to the pulp, and orientation of tubules may contribute to the lower predictable adhesion to dentin. Furthermore, adhesion could be influenced by other biological and clinical factors. These include the depth and permeability of the dentin, the tooth’s location in the mouth, the type of restorative material and procedure used, isolation, and the dentist’s experience [12, 13]. In the present study, the ISO/TS 11405:2016 protocol was followed to standardize the laboratory procedures. Human extracted posterior caries-free and unrestored teeth with sound superficial dentin were selected due to their higher permeability of the dentin structure. Furthermore, one single operator performed all cementation procedures according to the manufacturer’s recommendations, a step that might minimize the technique sensitivity of the test materials.

### Limitations

In the present study, only two resin cement systems were evaluated. Consequently, the results are limited to these specific products. Ideally, including more resin cement systems would be preferable to determine if any further differences exist. The choice of cements was based on the fact that they are both recommended for use with zirconia, yet they have some interesting differences. The Panavia V5 cement system has specific primers indicated for zirconia, and the manufacturer states that it does not recommend using its primers with other composite cements, even those within its own brand, while the RelyX Ultimate cement system offers a simple, universal primer designed for multipurpose use.

The cementation procedures were performed by a single operator. This aspect might be viewed as a limitation in terms of the study’s generalizability, where involving multiple operators would typically be preferred. However, in the case of an experimental in vitro study, it is considered a strength, because the primary study aim was to evaluate the effect of combining different primers on bond strength rather than the technique sensitivity of these materials.

Based on the results of this study, the Panavia V5 cement system is preferable for bonding to zirconia, especially in clinical situations where restorations rely more on adhesive bonding than on macromechanical retention. Moreover, this study highlights the potential effects—both negative and positive—of combining primers and cements from different cement systems, which could lead to less predictable clinical outcomes. However, caution should be taken when interpreting laboratory results, as the bond strength values observed cannot be directly applied to the clinical situations [24].

Future research, including other types of cement systems, and possibly including different surface pretreatments, is needed. Although microscopic analysis is sufficient to evaluate failure mode, including a scanning electron microscope (SEM) in future studies could be interesting and provide a more detailed analysis.

## Conclusion

Within the limitations of this current study, it may be concluded that:

1. Bond strength between zirconia and dentin was superior when using all primers from only the Panavia V5 system, regardless of whether the cement was RelyX Ultimate or Panavia V5.
2. The combination of primers in cement systems from different brands may significantly affect the bonding effectiveness. Therefore, using products within a single cement system line and following the manufacturer’s recommendations for indications and use should be the primary consideration.

## Data Availability

The data supporting the findings of this study can be obtained from the corresponding author, ML, upon reasonable request.
